# Genomic differentiation of three pico‐phytoplankton species in the Mediterranean Sea

**DOI:** 10.1111/1462-2920.16171

**Published:** 2022-08-24

**Authors:** Ophélie Da Silva, Sakina‐Dorothée Ayata, Enrico Ser‐Giacomi, Jade Leconte, Eric Pelletier, Cécile Fauvelot, Mohammed‐Amin Madoui, Lionel Guidi, Fabien Lombard, Lucie Bittner

**Affiliations:** ^1^ Sorbonne Université, CNRS, Laboratoire d'Océanographie de Villefranche, LOV Villefranche‐sur‐Mer France; ^2^ Institut de Systématique, Evolution, Biodiversité (ISYEB), Muséum national d'Histoire naturelle, CNRS, Sorbonne Université, EPHE, Université des Antilles Paris France; ^3^ Sorbonne Université, UMR 7159 CNRS‐IRD‐MNHN, LOCEAN‐IPSL Paris France; ^4^ Department of Earth, Atmospheric and Planetary Sciences Massachusetts Institute of Technology Cambridge Massachusetts USA; ^5^ Génomique Métabolique, Genoscope Institut François Jacob, CEA, CNRS, Univ Evry, Université Paris‐Saclay Evry France; ^6^ Research Federation for the Study of Global Ocean Systems Ecology and Evolution, FR2022/Tara Oceans GOSEE Paris France; ^7^ Institut de Recherche pour le Développement (IRD), UMR ENTROPIE Nouméa New Caledonia; ^8^ Service d'Etude des Prions et des Infections Atypiques (SEPIA), Institut François Jacob, Commissariat à l'Energie Atomique et aux Energies Alternatives (CEA), Université Paris Saclay Fontenay‐aux‐Roses France; ^9^ Institut Universitaire de France (IUF) Paris France

## Abstract

For more than a decade, high‐throughput sequencing has transformed the study of marine planktonic communities and has highlighted the extent of protist diversity in these ecosystems. Nevertheless, little is known relative to their genomic diversity at the species‐scale as well as their major speciation mechanisms. An increasing number of data obtained from global scale sampling campaigns is becoming publicly available, and we postulate that metagenomic data could contribute to deciphering the processes shaping protist genomic differentiation in the marine realm. As a proof of concept, we developed a findable, accessible, interoperable and reusable (FAIR) pipeline and focused on the Mediterranean Sea to study three a priori abundant protist species: *Bathycoccus prasinos*, *Pelagomonas calceolata* and *Phaeocystis cordata*. We compared the genomic differentiation of each species in light of geographic, environmental and oceanographic distances. We highlighted that isolation‐by‐environment shapes the genomic differentiation of *B. prasinos*, whereas *P. cordata* is impacted by geographic distance (i.e. isolation‐by‐distance). At present time, the use of metagenomics to accurately estimate the genomic differentiation of protists remains challenging since coverages are lower compared to traditional population surveys. However, our approach sheds light on ecological and evolutionary processes occurring within natural marine populations and paves the way for future protist population metagenomic studies.

## INTRODUCTION

Single‐celled eukaryotes or protists are major contributors to the diversity of plankton in the oceans (de Vargas et al., 2015; Moon‐van der Staay et al., 2001). They encompass a myriad of lifestyles, trophic modes, as well as morphological characteristics (Caron et al., 2012, 2017) and play key roles in the functioning of marine pelagic ecosystems, impacting trophic dynamics and global biogeochemical cycles (Biard et al., 2016; Gasol & Kirchman, 2018). Protists have long been under‐explored especially from a genomic point of view (Del Campo et al., 2014; Sibbald & Archibald, 2017). The scarcity of reference genomic data for protists results in a misunderstanding of the processes that underpin the contemporary distribution of genetic diversity in natural populations (Lebret et al., 2012; Logares, 2011). Protists are supposed to have vast population sizes and the potential for long‐distance dispersal (Dolan, 2005; Watts et al., 2013), which results in reduced evolutionary diversification processes (Finlay, 2002). In comparison with most macro‐organisms, protists are thus expected to have little opportunity for allopatric divergence and could show low levels of spatial genetic structure. Studies from the last decade, based on high‐throughput sequencing from natural communities via metabarcoding, have unveiled a high diversity of protist species, revealing both endemic and cosmopolitan species (Bittner et al., 2013; Forster et al., 2015; Logares et al., 2014; Malviya et al., 2016). Moreover, the very few genomic studies based on protist ‘model micro‐organisms’ such as *Emiliana huxleyi* (Read et al., 2013) or *Ostreococcus tauri* (Blanc‐Mathieu et al., 2017) highlighted a large intraspecific diversity in marine ecosystems. Consequently, even if marine protists have the potential for high dispersal through the currents, protist population structure has been frequently described at local (Evans et al., 2005), regional (Casteleyn et al., 2009) and even at global (Casteleyn et al., 2010) scales, and several processes were reported as drivers of their diversification (Sjöqvist et al., 2015).

In the marine realm, gene flow among planktonic populations can be driven by marine currents, abiotic (i.e. physico‐chemical) environmental conditions as well as biotic factors. Oceanographic currents support directional dispersal, conditioning the physical connectivity between distant populations patterns (Godhe et al., 2013; Riginos et al., 2016). They have been identified as major drivers for the structuring of marine populations (Casabianca et al., 2012; Nagai et al., 2007). The asymmetric migration patterns associated could additionally favour local adaptation (Kawecki & Holt, 2002; Sjöqvist et al., 2015). Genetic differentiation could also be driven by natural selection through environmental conditions such as silicate and nitrate/nitrite concentrations (Gao et al., 2019), or changes in salinity (Godhe et al., 2016; Sjöqvist et al., 2015), light or temperature (Latorre et al., 2021; Mena et al., 2019).

To date, population genetic studies have focused on a restricted number of protist species and on sparse genomic markers. With the expansion of high‐throughput sequencing, single nucleotide polymorphisms (SNPs) analysis is emerging as a powerful approach to infer population genetic differentiation among natural populations. SNPs are abundant, randomly distributed in genomes, and show low mutation rates and low false genotyping rates, while representing fair statistical power (Selkoe et al., 2016). SNP detection methods consist in mapping short sequences (reads) obtained by high‐throughput sequencing on longer reference sequences. Recent studies started to provide a metagenome‐level description of the ecological preferences for a few protists (Leconte et al., 2020; Seeleuthner et al., 2018; Vannier et al., 2016) and one of them analysed proxies of species obtained from a genomic reference‐free method (metavariant species; Laso‐Jadart et al., 2021). However, to our knowledge, genetic differentiation of protists from metagenomes has not been explored in a systematic way and there are no guidelines for the implementation of the ecological and evolutionary processes that are shaping their diversity at the species scale.

The objective of our study was to develop an original bioinformatics pipeline aiming to exploit the currently available metagenomic data for the characterization of genomic differentiation of protists in the marine ecosystems. To address this issue, we focused on the Mediterranean Sea, which is an ideal location to study population genomics (i.e. a semi‐enclosed marginal sea characterized by tortuous coastlines, with several environmental gradients despite a highly dynamic circulation, Ayata et al., 2018), on three a priori abundant planktonic protistan species in this area (de Vargas et al., 2015). We gathered reference sequences and metagenomic data previously published for which genomic differentiation could be highlighted at the species scale and tentatively explained by external drivers, such as geography, environmental conditions and oceanographic circulation. We hypothesized that genomic differentiation is greater among distant populations (Wright, 1943) and/or among populations sampled in different hydrological environments (Wang & Bradburd, 2014). We obtained contrasted results for all three species, which allowed us to discuss how current metagenomic data could support and provide new resources for population genomics studies for overlooked but abundant and ecologically relevant organisms.

## EXPERIMENTAL PROCEDURES

Our global analysis strategy for population genomics based on metagenomic data is summarized in Supporting Information S8 and all scripts and data are openly available on https://github.com/opheliedasilva/popmetag.

### Selection of protist species for the study of genomic populations

In order to study genomic differentiation within marine protist populations, we chose to exploit metagenomic data collected in the Mediterranean Sea during the Tara Oceans (TO) expedition (Alberti et al., 2017), in which protists prevailed from pico‐ to microplankton size fractions (i.e. from 0.8 up to 180 μm). We first analysed the abundance of the V9 eukaryotic metabarcodes in the TO Mediterranean samples (de Vargas et al., 2015) to identify dominant taxa over all stations for which a genomic/transcriptomic reference was available (Supporting Information S1). From there, we selected three phylogenetically distinct planktonic species: *B. prasinos* (Eikrem & Throndsen, 1990), *P. calceolata* (Andersen et al., 1993) and *P. cordata* (Zingone et al., 1999). While transcriptomes were used for *P. calceolata* and *P. cordata* (RCC969 and RCC1383, respectively), a reference genome was used for *B. prasinos* (RCC1105). The high gene density of *B. prasinos* genome (Moreau et al., 2012) was assumed to limit the impact of intergenic regions in the analysis (Supporting Information S7). Given the size range of these organisms, we focused on the 0.8–5 μm size fraction in the TO data, consisting of 13 metagenomics samples available from the surface layer (accession number PRJEB4352, Carradec et al., 2018; Supporting Information S1), containing on average 185 million sequence reads.

We built a bioinformatic pipeline to extract single nucleotide polymorphisms (SNPs) from metagenomic sequences in comparison to reference sequences (here genome or transcriptome). It consisted of five steps (detailed in the Supporting Information S2, and the whole bioinformatic pipeline is available on GitHub ): (1) checking the quality of the metagenomic reads to remove the low‐quality ones (Trimmomatic; Bolger et al., 2014), (2) mapping the metagenomic reads on the reference sequences to pull out reads of the targeted species (bwa mem; Li, 2013), (3) filtering aligned reads, first to remove low complexity sequences and avoid spurious alignments (PRINSEQ; Schmieder & Edwards, 2011) and second to reduce the recruitment of reads from a closely related species (reads aligned with less than 95% identity were removed; Vannier et al., 2016), (4) detecting genomic variants in comparison with the reference sequence, and (5) filtering the variants in order to only keep the SNPs (e.g. indels were removed). To minimize false positives, SNPs were filtered based on their vertical coverage (i.e. mean number of reads aligned at each position of the assembly): we only kept SNPs supported by at least four reads but less than *μ* + 2*σ* of vertical coverage (*μ* is the mean and *σ* is the standard deviation of SNP vertical coverage, in order to remove SNPs also abundant in closely related species). The output of our pipeline corresponded to an abundance table of the SNPs in each station. Based on this output, we computed for each SNP the frequency of alleles in each station. *F*_ST_ were calculated for each SNP at each pair of stations. As the number of allelic frequencies greatly varied from one locus to another, average frequency used in *F*_ST_ calculation was always computed for the two stations considered and not for all of them. Finally, the genomic differentiation between each pair of stations (pairwise *F*_ST_) corresponded to the median *F*_ST_ and was used as the genomic distance between populations. For *B. prasinos*, 20 pairs of stations had no shared SNP and therefore no genomic differentiation was computed for these pairs of stations. For *P. calceolata*, one station had no common SNPs with all the others and was therefore removed from the analysis. For each species, the global *F*_ST_ was computed as the mean pairwise *F*_ST_. For each species, we created heatmaps to visualize genomic distances between pairs of stations (Figure 5). Dendrograms, built by hierarchical clustering (complete linkage), were added on the heatmaps to help identifying groups of stations genetically close. The missing genomic distances (20 values out of 78 for *B. prasinos*) have been replaced by the mean value to perform the clustering.

### Calculations of geographic, environmental and oceanic distances

Firstly, the latitude and longitude of sampling (metadata from PANGAEA; Pesant et al., 2015) were used to compute geographic distances among pairs of stations (i.e. minimal distances between two stations without crossing the lands). Secondly, each TO sample was associated with its hydrological and biogeochemical environment based on geographic coordinates and depth (TO metadata, Pesant et al., 2015). The environmental variables extracted from Medatlas‐II climatologies (Fichaut et al., 2003) included surface temperature (°C), surface salinity (PSU) and surface concentrations of ammonium (mmol.m^−3^), oxygen (ml.l^−1^), nitrate (mmol.m^−3^), nitrite (mmol.m^−3^), phosphate (mmol.m^−3^), silicate (mmol.m^−3^) and chlorophyll a (mg.m^−3^). A PCA was performed on these normalized and standardized variables (Legendre & Legendre, 2012) for a total of 22 TO stations (13 stations corresponding to our metagenomic sample and nine additional TO stations). The environmental distances between pairs of stations were computed as their Euclidean distances in the PCA space (using only significant axes based on the Kaiser–Guttman criterion) and the stations were clustered using hierarchical clustering. Thirdly, physical transport by ocean circulation was estimated with Lagrangian model simulations to compute oceanographic distance between stations. As no assumption about mechanisms of dispersal and underlying model settings for each organism could be established, data from two types of existing models were used to assess Lagrangian transport in the Mediterranean Sea. The first Lagrangian dataset was a product of Berline et al. (2014) providing the mean connection time (MCT, in days) for each pair of stations. The second dataset was obtained by performing simulations of the model of Ser‐Giacomi et al. (2015) based on the Lagrangian flow network approach. The dataset corresponded to a connectivity matrix estimating the probability of connection (PC) for a particle leaving a station to end up in another station in a given time period. To estimate PC over time, three dispersal durations were used (3, 6, and 12 months) and averaged to integrate dispersal characteristics at various temporal scales (seasonal, biannual, and annual circulation). As Lagrangian matrices are asymmetric, we chose the maximum PC and the minimum MCT as oceanographic distance for each pair of stations. More details about Lagrangian datasets are provided in Supporting Information S5.

### Statistical analyses

The links between genomic distances and geographic, environmental and oceanographic circulation constraints were assessed through linear regressions using the following model:

$$y=\beta_{0}+\beta_{\mathrm{geo}}\;x_{\mathrm{geo}}+\beta_{\mathrm{env}}\;x_{\mathrm{env}}+\beta_{\mathrm{pc}}\;x_{\mathrm{pc}}+\beta_{\mathrm{mct}}\;x_{\mathrm{mct}}+\varepsilon$$

where *y* corresponds to the normalized genomic distances *F*_ST_/(1−*F*_ST_), *β*_0_ is the intercept coefficient (i.e. predicted response when pairs of stations are not distant in terms of geography, environment or oceanographic circulation). The geographic (*β*_geo_), environmental (*β*_env_) and oceanographic (*β*_pc_ and *β*_mct_) coefficients quantify the effects of geographic, environmental and oceanographic distances (respectively *x*_geo_, *x*_env_, *x*_pc_ and *x*_mct_) on the genomic distances. *ε* is the error term (i.e. random component between the variable to explain and the explanatory variable). For each species, we selected the optimal model by an exhaustive search procedure using the Bayesian information criterion (BIC). Fisher tests (null vs. selected model) were carried out to test the overall significance of the linear models. Variables impacting genomic differentiation were identified with Student tests using a threshold of 0.05.

All analyses were conducted in R (v3.5.0; R Core Team, 2020) using the packages: tidyverse (v1.2.1; Wickham et al., 2019), ggreprel (v0.8.1; Slowikowski, 2020), ggpubr (v0.3.0; Kassambara, 2020), maps (v3.3.0; Becker et al., 2018), and viridis (v0.5.1; Garnier, 2018) for graphs and maps; and FactoMineR (v1.42; Lê et al., 2008); gdistance (v1.3; van Etten, 2017), and leaps (v3.0, Lumley & Miller, 2013) for statistical analysis.

## RESULTS

### Genomic distances from metagenomic samples based on a selection of three protist species

**FIGURE 1:**
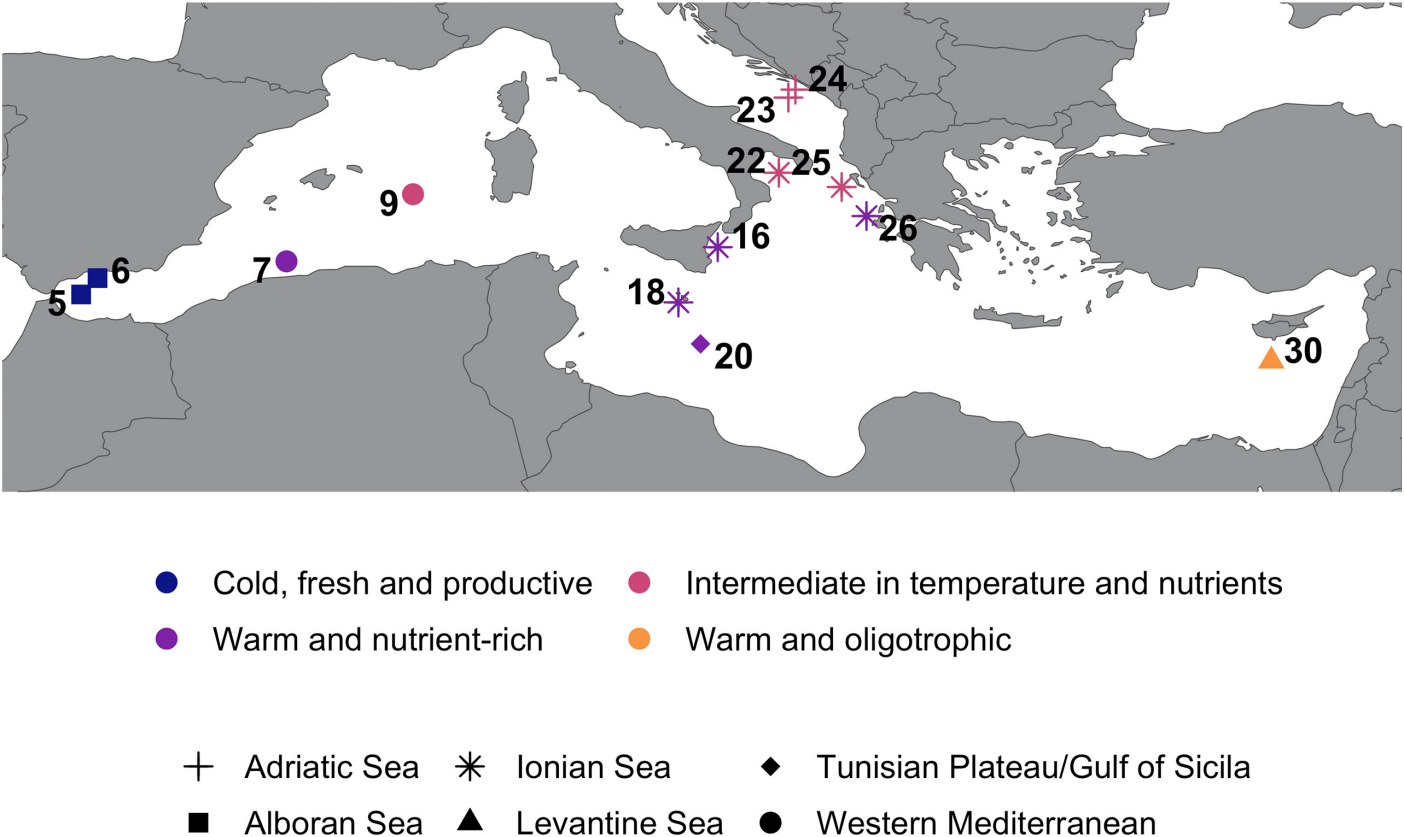
Geographic location of the 13 stations sampled during the Tara oceans for metagenomic analysis. Stations are indicated by numbers (with increasing numbers from west to east, following the Tara oceans cruise trajectory). Geographic entities are based on the marine ecoregions of the world (Spalding et al., 2007) and represented by different shapes. Environmental entities determined through principal component analysis (PCA) are indicated by colours (defined in Figure 4).

**FIGURE 2:**
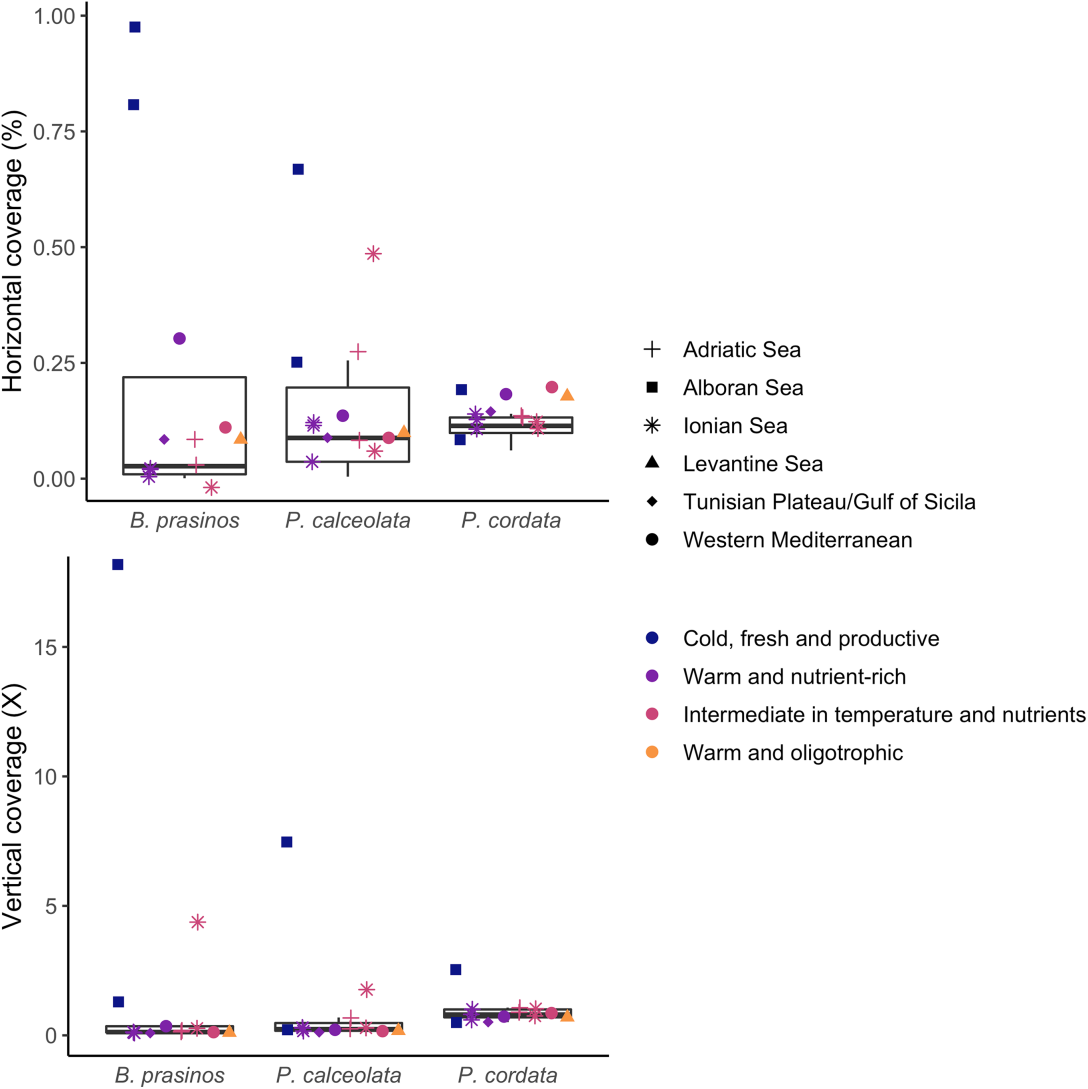
Distributions of horizontal and vertical coverages for each species within the 13 Mediterranean Sea stations (i.e. percentage of reference covered by at least one read and mean number of reads aligned at each position of the reference, respectively). Type of shape: geographic entities (defined in Figure 1). Colours: environmental entities (defined in Figure 4).

The genomic distances were computed for three phylogenetically distinct planktonic species (Supporting Information S1): *Bathycoccus prasinos* (Chlorophyta; Eikrem & Throndsen, 1990), *Pelagomonas calceolata* (Ochrophyta; Andersen et al., 1993) and *Phaeocystis cordata* (Haptophyta; Zingone et al., 1999). These three species are widespread at global scale, with an ubiquitous distribution for *B. prasinos* (Moreau et al., 2012; Vannier et al., 2016) and *P. calceolata* (Worden et al., 2012), or present in many areas for *P. cordata* (i.e. Red Sea, Indian Ocean, Mediterranean Sea; Decelle et al., 2012). *B. prasinos* is a major contributor of the primary production and shows a seasonal cycle in the Mediterranean Sea (Lambert et al., 2019; Moreau et al., 2012). *P. calceolata*, involved in nitrate assimilation (Dupont et al., 2015), has been overlooked in the Mediterranean Sea. Finally, *P. cordata* has been detected in free‐living mode and in symbiosis with Acantharia in the Mediterranean Sea (Decelle et al., 2012). A reference genome was selected for *B. prasinos* (Moreau et al., 2012), whereas, at the time of our analysis, only transcriptomes were publicly available for *P. calceolata* and *P. cordata* (Johnson et al., 2019; Keeling et al., 2014). The cumulative length of these assemblies varied by twofold from *P. cordata* to *P. calceolata* (respectively, 9.4 and 21 Mb), while the length of *B. prasinos* assembly was intermediate (15 Mb). Based on the cell size of these organisms (Supporting Information S1), we focused on the 0.8–5 μm size fraction of the Tara Oceans (TO) dataset, consisting of 13 metagenomic samples collected at the surface layer from 13 stations in the Mediterranean Sea (Figure 1, accession number PRJEB4352, Carradec et al., 2018; see Supporting Information S2 for details). We mapped the metagenomic reads (on average 185 million reads/sample) on reference assemblies and obtained horizontal and vertical coverages between species (Figure 2; percentage of the reference covered by at least one read and mean number of reads aligned at each position of the reference, respectively). *B. prasinos* and *P. calceolata* displayed higher horizontal coverages than *P. cordata* despite their longer reference sizes (maximal horizontal coverage, *B. prasinos*: 98.1%, *P. calceolata*: 68.2%, *P. cordata*: 19.6%). The mean vertical coverage was also more heterogeneous for *B. prasinos* and for *P. calceolata* than for *P. cordata* (min–max mean vertical coverage, *B. prasinos*: 0.085–17.6 X, *P. calceolata*: 0.119–7.2 X, *P. cordata*: 0.51–2.47 X). *B. prasinos* showed highest coverages at stations 5 and 6, located in the Western part of the Mediterranean Sea (Alboran Sea).

SNPs were detected from aligned and filtered reads (Experimental procedures, Supporting Information S2). *B. prasinos*, *P. calceolata* and *P. cordata* showed different total numbers of SNPs over reference size ratio (respectively, 3.4, 51.4 and 0.5 SNPs/Mb). In average, only 9.26%, 9.67% and 18.74% of the total number of SNPs were observed in each station for *B. prasinos*, *P. calceolata* and *P. cordata*, respectively. Moreover, a filtering on the vertical coverage led to the removal of SNPs (i.e. SNPs having between 4 and μ + 2σ vertical coverage were kept; Experimental procedures, Supporting Information S2). Consequently, pairwise *F*_ST_ (i.e. median genomic distance as defined in the Wright's formulation, where 0 indicates no genomic differentiation and 1 means maximal genomic differentiation) between all station pairs were computable for *P. cordata*, whereas for *P. calceolata*, the station 30 had to be removed, and for *B. prasinos* 20 pairwise *F*_ST_ were not computable. We obtained 78 pairwise genomic distances (pairwise *F*_ST_) for *P. cordata*, 66 for *P. calceolata* and 58 for *B. prasinos*.

**FIGURE 3:**
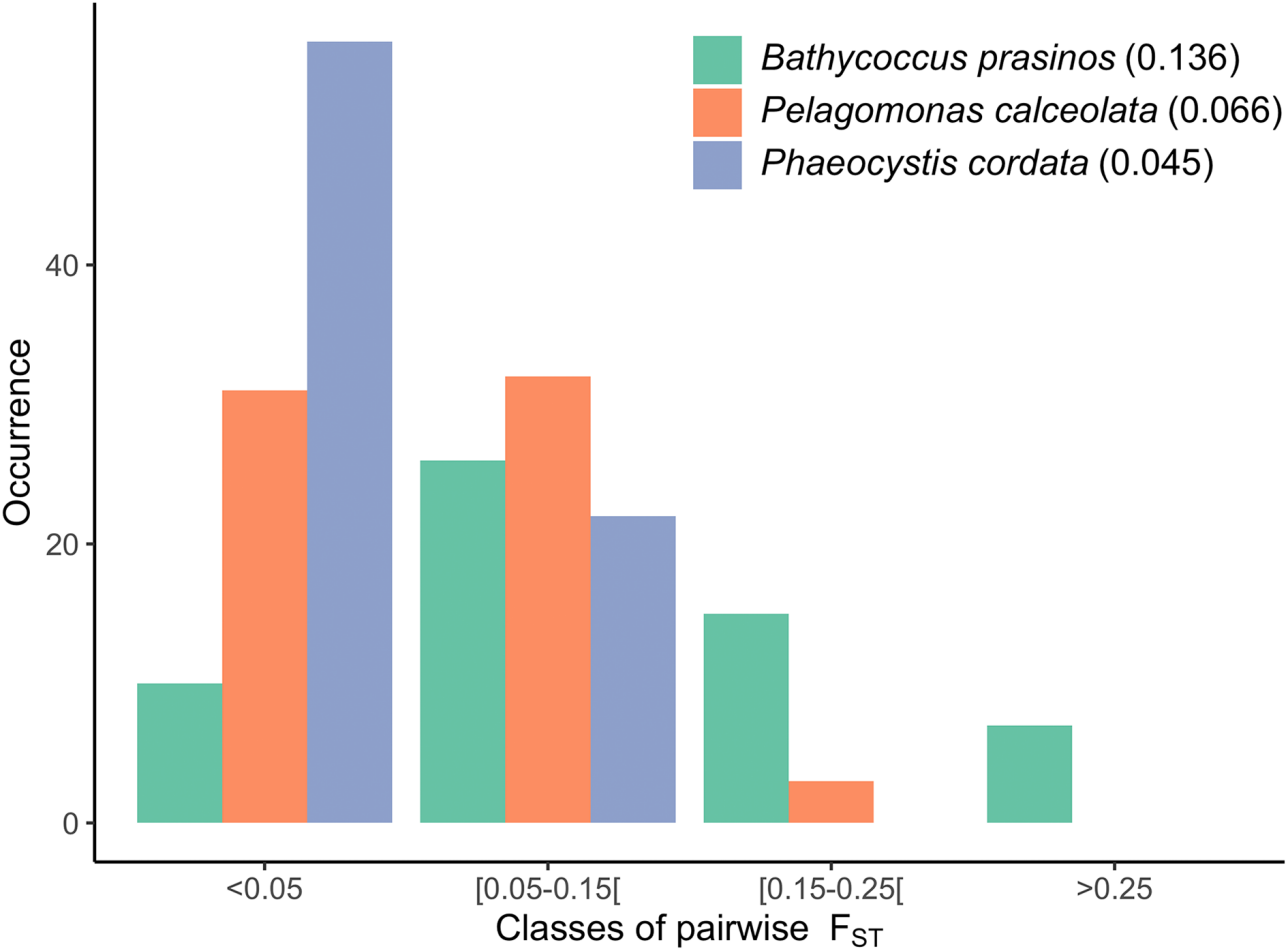
Distribution the genomic distances for each species between the 13 Mediterranean Sea stations. The pairwise *F*_ST_ were grouped following Hartl and Clark (1997) by four classes of genomic differentiation (<0.05: little, [0.05–0.15]: moderate, [0.15–0.25]: high, >0.25: very high).

Our results show that the Mediterranean Sea populations of *B. prasinos* had a stronger global genomic differentiation (global *F*_ST_ = 0.136) than *P. calceolata* (global *F*_ST_ = 0.066) and *P. cordata* (global *F*_ST_ = 0.045) (Figure 3). *B. prasinos* also showed the most contrasted genomic differentiation (pairwise *F*_ST_) ranging from little (0.012) to very high (0.476) (Figure 3). *B. prasinos* was also the only species showing very high genomic differentiation values (seven *F*_ST_ values > 0.25). *P. calceolata* showed little to high genomic differentiation (*F*_ST_ ranging from 0.019 to 0.181), whereas *P. cordata* had the lowest *F*_ST_ values (ranging from 0.026 to 0.069). The maximum *F*_ST_ for *P. cordata* was observed in the little genomic differentiation class, whereas *P. calceolata* and *B. prasinos F*_ST_ peaked in the moderate genomic differentiation class (Figure 3).

### Geographic, environmental and oceanographic distances between stations

Geographic distances were computed as the minimal distances between each pair of stations, and ranged between 33.55 and 3624.49 km (mean: 1255.13 km; Figure 1, Supporting Information S3).

**FIGURE 4:**
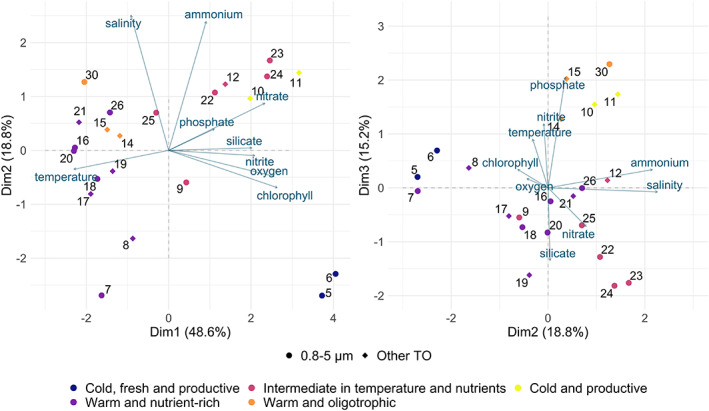
Environmental characterization of the Mediterranean Sea stations. Principal component analysis (PCA) of the environmental variables retrieved at 22 stations indicated by points and numbers. The 13 TO stations studied, reported with dots, and 9 extra stations are reported with diamonds. Colours of the points indicate the environmental clusters the stations belong to (hierarchical clustering). Environmental variables are represented with blue arrows. The significant axes (Dim1, Dim2, Dim3) are presented with their corresponding percentages of variance.

A principal component analysis (PCA) was performed on the environmental conditions of the stations, in order to infer environmental distances between each pair of stations. To strengthen the statistics, we analysed the 13 stations corresponding to our metagenomic samples and nine additional TO stations also sampled in the Mediterranean Sea (Figure 4). Three significant axes were identified: the first PCA axis (Dim1, 48.6% of the total variance) distinguished the warmer and more oligotrophic stations (Dim1 < 0) from the colder and nutrient richer ones (Dim1 > 0); the second PCA axis (Dim2, 18.8% of the total variance) separated the saltier and ammonium‐rich stations (Dim2 > 0) from the less salty and ammonium‐poor stations (Dim2 < 0); and the third PCA axis (Dim3, 15.2% of the variance) divided the silicate‐rich stations (Dim3 < 0) from the phosphate and nitrite‐rich ones (Dim3 > 0). Environmental distances were calculated as the variance‐weighted Euclidean distances on the first three dimensions (Supporting Information S4).

Oceanographic distances were inferred from Lagrangian modelling both as the mean connection times (data from Berline et al., 2014) and as connection probabilities among stations (Ser‐Giacomi et al., 2015). They ranged between 46.15 and 545.9 km (mean: 219.9 km) and 1.72 × 10^−7^ and 9.07 × 10^−3^ (mean: 5.98 × 10^−4^), respectively (Supporting Information S5).

### Deciphering drivers of genomic differentiation

For each of the three planktonic protist species, the link between genomic differentiation and geography, environment or oceanographic circulation was assessed through linear regression models. We used Fisher tests to show that our linear models explain the genomic differentiation of *B. prasinos* and *P. cordata* better than the null models (Table 1). In contrast, our model did not provide a better fit than the null model for *P. calceolata*. The optimal selection model procedure led to the selection of environmental distances for *B. prasinos* and of geographic distances for *P. cordata* (Table 1). Selected models explained 15.24% of the genomic differentiation for *B. prasinos* and 12.04% for *P. cordata*. The genomic differentiation of *B. prasinos* significantly increased with environmental distances, whereas the genomic differentiation of *P. cordata* significantly increased with geographic distances.

**TABLE 1:** Results of statistical models testing the impact of geography, environment, and ocean circulation on genomic distances of three planktonic species

Species	Selected versus null model	Selected model	*R* ^2^
*Bathycoccus prasinos*	*p* value = 0.0025	*β*_env_ = 0.0382 *p* value_env_ = 0.0025	15.24
*Pelagomonas calceolata*	*p* value = 0.344		
*Phaeocystis cordata*	*p* value = 0.0019	*β*_geo_ = 4.624 × 10^−6^ *p* value_geo_ = 0.0019	12.04

*Note*: The *p* value of the overall significance test is provided (selected vs. null model). For each selected model, the value of the regression coefficient *β* and the associated *p* values are indicated for the selected variables (env: Environmental distances, geo: Geographic distances), as well as the determination coefficient *R* ^2^.

**FIGURE 5:**
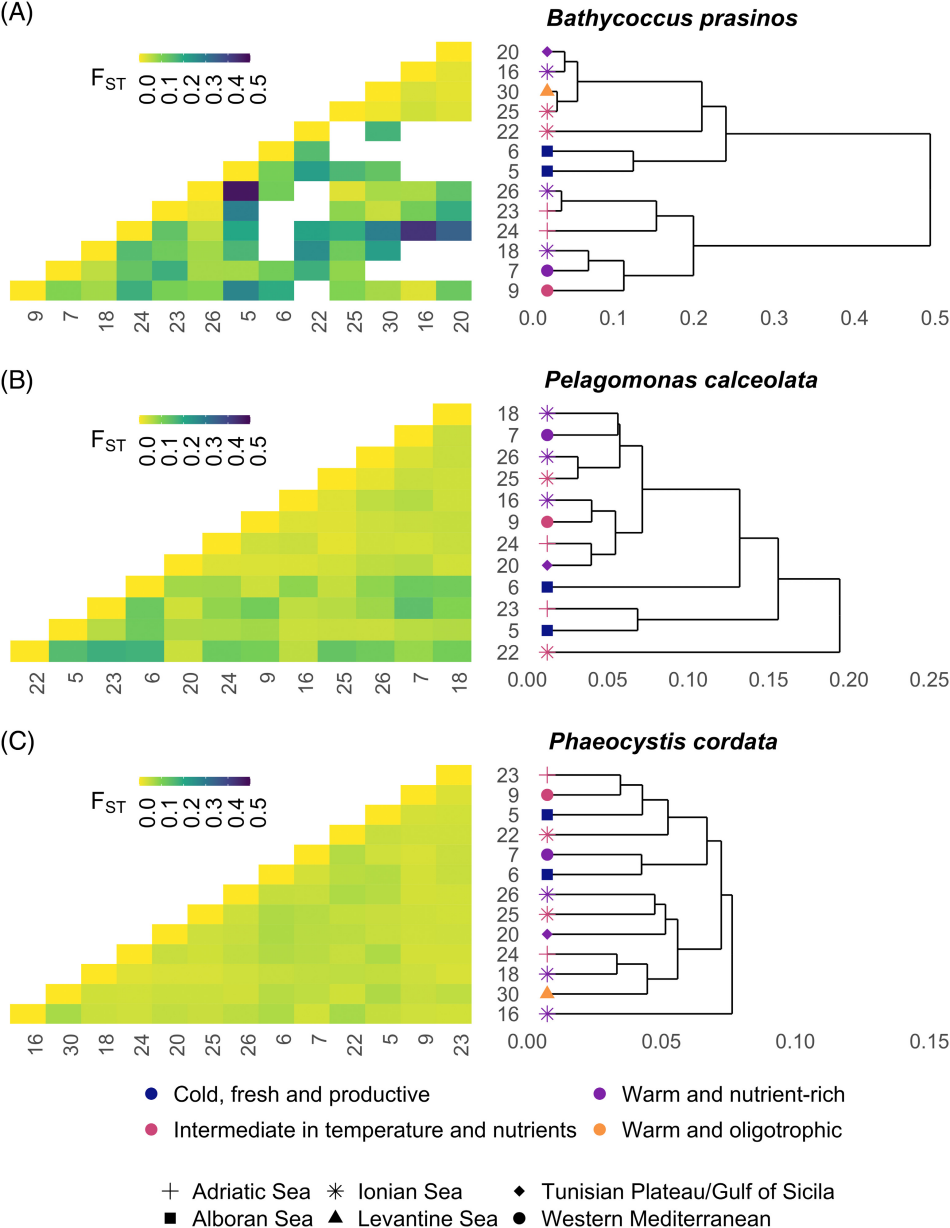
Genomic differentiation among the Mediterranean Sea populations. Heatmaps of genomic distances (pairwise *F*_ST_) with associated dendrograms obtained by hierarchical clustering for (A) *B. prasinos*, (B) *P. calceolata* and (C) *P. cordata*. For *B. prasinos*, missing values (due to absence of common SNPs) were replaced by the mean value of *F*_*ST*_ of the stations. Type of shape: geographic entities (defined in Figure 1). Colours: environmental entities (defined in Figure 4).

The highest genomic differentiations of *B. prasinos* were observed between stations 5 (Alboran Sea) and 26 (Ionian Sea) and between stations 16 and 24 (Ionian Sea and Adriatic Sea, respectively; Figures 4 and 5A), whereas the smallest genomic differentiations were observed among stations 16, 20, 25 and 30, all located in the Eastern basin of the Mediterranean Sea. This group of stations was genetically distant from stations 23 and 24 (Adriatic Sea), from stations 5 and 6 (Alboran Sea) and also from station 22 (Ionian Sea) (Figure 5A). These two groups of stations (16, 20, 25, 30 vs. 5, 6, 22, 23, 24) were environmentally separated by the nutrient‐temperature gradient (Dim1, Figure 4), with the former stations characterized by warmer and more oligotrophic conditions (Eastern Basin of the Mediterranean Sea).

For *P. cordata*, the highest genomic differentiations were observed between stations from the Western Basin (stations 5, 6, 7, 9, Alboran Sea and Western Mediterranean) and from the Eastern Basin (stations 18, 20, 24, 25, 26, 30, Ionian Sea, Tunisian Plateau/Gulf of Sicilia, Adriatic Sea and Levantine Sea) (Figures 1 and 5B). Exceptions were however observed, with spatially close stations that show high genomic differences (e.g., stations 9 and 23, or 18 and 24), confirming that geographic distances are not the only factor driving the genomic differentiation of this species.

## DISCUSSION

Our population genomics approach based on metagenomic data and applied to marine planktonic protists offers new insights into the genomic differentiation of understudied organisms and describes the main abiotic drivers shaping it.

### Main challenges for population genomics approach based on metagenomics

In this study, we developed a computational method in order to apply population genetic concepts from metagenomic data. This pipeline was designed according to the FAIR principles (Wilkinson et al., 2016) and it can be transposed to any protist lineages as long as reference sequences are available. The number of species that can be investigated with this pipeline is thereby limited by the number of available reference assemblies. Historically, sequencing projects prioritized species of biomedical and biotechnological interest, or species that were easy to cultivate. This has led to strong biases in public molecular databases in which protist reference sequences are under‐represented (Del Campo et al., 2014; Keeling & Del Campo, 2017; Sibbald & Archibald, 2017). In the last decade, an increasing number of sequencing initiatives ha tried to cope for this limitation (Keeling et al., 2014), but most protist lineages remain unrepresented in public databases (e.g. about 200 genomes in the Genome Online Database in February 2017, mostly parasitic; Sibbald & Archibald, 2017). So far, only a few dozen species are available for a focused analysis of the Mediterranean Sea. It is, however, very likely that the increasing availability of reference sequences will allow population genomics' analyses based on metagenomic data in the near future.

Based on metabarcoding data, our three targeted species were potentially abundant in the Mediterranean Sea (Supporting Information S1). However, the reference sequences selected may include biases, as references do not come from the exact same geographic location as the metagenomic data. The *P. calceolata* reference strain (RCC969) was sampled in the southern Pacific Ocean (Lê et al., 2008), which is, as the Mediterranean Sea, an oligotrophic area. However, variations among *Pelagomonas* strains have already been reported, notably driven by different light‐acclimation strategies (Kulk et al., 2012; Worden et al., 2012). The two other references, *P. cordata* (RCC1383) and *B. prasinos* (RCC1105), were both isolated in the Mediterranean Sea, respectively, from the Tyrrhenian Sea (Zingone et al., 1999) and from Banyuls' Bay (north‐western Mediterranean Sea; Moreau et al., 2012), but our 13 metagenomic samples were not collected from these areas. The relatively low mapping coverages obtained might reflect the fact that dominant lineages in the natural communities differ significantly from the current reference lineages. Reference lineages come mainly from species in culture or more rarely from species that have been isolated locally from the environment (e.g. single amplified genomes; Del Campo et al., 2014). Therefore, references are to date poorly indicative of the genomic variability in natural populations (Bittner et al., 2013; Laso‐Jadart et al., 2021; Worden et al., 2012). Using metagenomic‐assembled genomes (MAGs) as new references can partly circumvent this issue, because they correspond to abundant biological units in the studied ecosystems. MAGs have been recently built for microbial eukaryotes, either from metagenomes (Delmont et al., 2022) or from metatranscriptomes (Vorobev et al., 2020). But, as they result from ‘consensus assemblies’, they can integrate the variability of several sampled organisms or populations, and the biological scale at which the study is carried out (species, genus, biological association/holobiont) remains uncertain (Shaiber & Eren, 2019). The representativity of current MAGs is high, but likely also far from the phylogenetic diversity highlighted by metagenomic studies based on classical de novo assemblies (Carradec et al., 2018 vs. Delmont et al., 2022). Highly abundant but genetically complex lineages (e.g. Dinoflagellates) still fail to be reconstructed from the environment, mainly due to the globally shallow sequencing depth generally achieved for environmental samples.

The main difference between traditional population genetics studies and our metagenomics‐based approach is that metagenomic data provides occurrences for the whole community. Hence, this imposes a first analytical procedure during which the sequences of targeted species have to be extracted from the bulk data. Even if the TO metagenomic samples were obtained by the filtration of large seawater volumes and were sequenced to a consequent depth (160 million reads per sample; Alberti et al., 2017), it has previously been shown that the sampling effort for the smallest planktonic fraction did not result in a saturation of the eukaryotic genes (Carradec et al., 2018). Our targeted species, even if theoretically abundant in the Mediterranean Sea, might thus be underrepresented in the metagenomic samples. Nonetheless, we assumed that the direct mapping of metagenomic reads on references (instead of their use through de novo assembly of long sequences) could depict the genomic structure of protist populations (Leconte et al., 2020; Vannier et al., 2016). Filtering parameters were tuned to ensure the detection of good quality variants of our targeted species. Firstly, during the read recruitment step, alignments with less than 95% mean identity were discarded in order to obtain a proper genome abundance estimate for the targeted species. This identity threshold is comparable with previous estimations based on Chlorophyta lineages (Blanc‐Mathieu et al., 2017; Leconte et al., 2020) and ensures that all reads recruited belong to the same species, despite intraspecific variation. Future new genomic references on Haptophyta and Ochrophyta will allow refining this filtering step. Secondly, defining a minimal coverage threshold was not straightforward. In studies based on model organisms (i.e. mainly human, mice, few others Metazoa, as well as pathogenic Eubacteria) at least 30 X vertical coverage thresholds are usually expected (Davide & Donati, 2017; Sims et al., 2014), while in our study vertical coverages ranged between 0.085 X and 17.6 X. However, low minimal coverage thresholds (e.g. 4 X) have already been used (e.g. on copepods; Madoui et al., 2017), allowing for first population genomic inferences based on metagenomic data for non‐model planktonic species. In addition, the coverage threshold was centred around the mean vertical coverage (maximal coverage threshold of μ + 2σ) leading to the removal of SNPs due to the read recruitment of closely related species (Madoui et al., 2017; Laso‐Jadart et al., 2020, 2021; Supporting Information S6). Therefore, the three picoeukaryotes well illustrate how far the coverages obtained based on metagenomics are from classical population genomics approaches. Low coverages should be interpreted with caution. Nonetheless, even if *F*_ST_ estimates are based on a restricted amount of data, we believe that our results are valuable for the observed trends for genomic differentiation from natural populations of protists.

### Protist genomic differentiation in the Mediterranean Sea and its different drivers

A large number of population genetics studies have demonstrated how dispersal and environment impact macro‐organisms' population genetic diversity in the Mediterranean Sea (e.g., the striped red mullet, Dalongeville et al., 2018). In contrast, only a few studies have focused on protists. Two studies used micro‐satellites data for two dinoflagellate species and highlighted the existence of a genetic structuring between the Atlantic Ocean and the Mediterranean Sea (Lowe et al., 2012) as well as through circulation patterns in the Mediterranean Sea (Casabianca et al., 2012). However, most seascape genetic studies rely on the study of pluricellular, macroscopic organisms (Selkoe et al., 2016). High‐throughput metagenomics now enable to investigate highly abundant components of the ecosystems (e.g. the *Oithona nana* copepod, Madoui et al., 2017; *Oithona similis*, Laso‐Jadart et al., 2020) involving more and more microbes (Delmont et al., 2019; Faure et al., 2021; Laso‐Jadart et al., 2021; Leconte et al., 2020; Seeleuthner et al., 2018; Vannier et al., 2016). By analogy, we conducted a comparative study of three picoeukaryotes. While a reference genome was used for *B. prasinos*, reference transcriptomes were used for *P. calceolata* and *P. cordata*. Even if that may impact the observed patterns and their direct comparisons, we hypothesized that the bias is limited due to the compact genome of *B. prasinos*, which is a peculiar characteristic of Mamiellales (Moreau et al., 2012; Supporting Information S7). Our study highlighted that *B. prasinos* (Chlorophyta), *P. calceolata* (Ochrophyta) and *P. cordata* (Haptophyta) exhibit distinct spatial patterns, forced by different external constraints. As our statistical models did not explain a large part of the genomic differentiation for *B. prasinos* and *P. cordata*, and none for *P. calceolata*, our results must be interpreted with caution. For *B. prasinos*, gene flow appeared stronger among populations from similar environments, which suggested an isolation‐by‐environment pattern (Wang & Bradburd, 2014). Several mechanisms may also explain the genetic differentiation due to environmental forcing, especially local adaptation, non‐random mating due to adaptation or phenotypic plasticity (Sexton et al., 2014). Nonetheless, Vannier et al. (2016) identified that environmental conditions such as temperature and light may influence the distribution of *Bathycoccus*. Our isolation‐by‐environment hypothesis is in line with this assumption and suggests that the environmental conditions might drive the genomic differentiation of this lineage. For *P. cordata*, gene flow decreased with geographic distances, supporting a hypothesis of isolation‐by‐distance for this species (Wright, 1943). Isolation‐by‐distance has frequently been suggested as an important driver of genomic differentiation for protists (Casteleyn et al., 2010; Demura et al., 2014; Nagai et al., 2007) and can act at different scales. Indeed, isolation‐by‐distance has been described as a driver of genomic differentiation for the diatom *Pseudo‐Nitschia pungens* at global (Casteleyn et al., 2010), regional (Casteleyn et al., 2009) and local scales (Evans et al., 2005). Finally, the spatial scale of our sampling impacts the detection of genomic isolation in our datasets. Dalongeville et al. (2018) assessed the importance of geographic distances at long‐distance spatial scales (>1000 km) and of dispersal constraints at shorter spatial scales (<1000 km) for structuring the genetic diversity of the red mullet fish in the Mediterranean Sea. For the Dinoflagellates, *Oxyrrhis marina* and *O. maritima*, Lowe et al. (2010) identified contrasted genetic structures between the Atlantic and the Mediterranean Sea subpopulations. Future studies could benefit from sampling at finer and higher resolution to better decipher processes shaping protist population differentiation in natural environments.

### Protist dispersal and oceanographic circulation

Since planktonic protists are supposed to have potential for long distance dispersal and large population sizes (Cermeño & Falkowski, 2009; Finlay, 2002), the circulation may not impact their genomic differentiation (Cermeño & Falkowski, 2009; Gibbons et al., 2013; Hellweger et al., 2014). However, *P. cordata* showed a pattern of isolation‐by‐distance at the scale of the Mediterranean Sea, which was surprisingly stronger with geographic distance rather than with oceanographic distance. Indeed, oceanographic distance better represents the asymmetric dispersal of plankton. We thus expected to better explain protists differentiation, in the Mediterranean Sea where several frontal structures (i.e. boundaries between distinct water masses) correspond to switches in plankton community composition (Ayata et al., 2018). In our study, the computed geographic distance was correlated with both circulation and environmental distance and could then integrate both pieces of information. As a consequence, isolation‐by‐distance and isolation‐by‐environment may indirectly contribute together to the genomic patterns observed for *P. cordata*. In addition, for the three species studied, the statistical models did not explain most of the genomic differentiation, suggesting that other parameters should also be tested in order to improve the prediction of genomic differentiation within each of our protist species. In particular, these parameters include historic factors, such as population size, and biotic factors, such as competition between *B. prasinos* and other Mamiellales (as questioned by Leconte et al., 2020) or free‐living versus symbiont states for *P. cordata* (Uwizeye et al., 2021).

## CONCLUSION

Metagenomic data represent an opportunity to improve knowledge on genomic differentiation of marine plankton, in particular for protists, which play a crucial role in ecosystem functioning but remain poorly investigated mainly due to difficulties to maintain them in culture. In this study, the genomic differentiation of three protists species with contrasted life history traits was characterized in the Mediterranean Sea based on both metagenomic samples and reference assemblies. Although relatively weak genomic differentiation was observed, we were able to identify distinct drivers explaining the observed patterns. Our results suggest that, at the scale of the Mediterranean Sea, *B. prasinos* differentiation was constrained by isolation‐by‐environment process, whereas *P. cordata* differentiation was constrained by isolation‐by‐distance process. These identified processes cannot be extrapolated to the global ocean or to another basin. Although several limitations still remain for the use of metagenomic data for population genomics studies, for example, the need of a reference assembly close to wild population, our results describe a promising approach for future studies targeting uncultured but abundant and ecologically important species.

## CONFLICT OF INTEREST

The author declares that there is no conflict of interest that could be perceived as prejudicing the impartiality of the research reported.

## Supporting information


**Appendix S1** Supporting Information

## Data Availability

Pipeline, analysis scripts and contextual data are available on GitHub https://github.com/opheliedasilva/popmetag. Genomic data are available on Zenodo https://zenodo.org/record/6434681#.Yv6ngXZByiM.
