# Supplementary material for: Genomic differentiation of three pico‐phytoplankton species in the Mediterranean Sea
Source: Environ Microbiol. 2022 Aug 24;24(12):6086–99. doi: 10.1111/1462-2920.16171 (PMC10087736; doi:10.1111/1462-2920.16171)
Supplement: Supplementary file 1 — Appendix S1 Supporting Information [file EMI-24-6086-s001.doc]

Supporting Information 1. Methods for preliminary investigations

Preliminary investigations were conducted to select relevant species for the Mediterranean Sea (*i.e.*, protist species for which enough data were available). The abundance of the V9 eukaryotic metabarcodes in the TO Mediterranean samples (de Vargas et al., 2015) were analysed to identify the dominant taxa over all stations. 6.8% of the taxa (*i.e.*, 580 distincts metabarcodes, with 439 species-level assignations) were present in at least 93.75% of the stations. In parallel, evidence for the presence of these presumably abundant protist species in the Mediterranean Sea were searched in the literature. Only planktonic (*i.e.*, benthic removed) protists were targeted. About twenty species were listed and among those, the availability of reference genome(s) or transcriptome(s) led us to select three phylogenetically distinct planktonic species (Figure S1, Table S1): *Bathycoccus prasinos* (Eikrem and Throndsen, 1990), *Pelagomonas calceolata* (Andersen et al., 1993) and *Phaeocystis cordata* (Zingone et al., 1999).

## We also considered using MAGs/SAGs as reference, in particular for *B. prasinos* for which the available references are of good quality (Delmont et al., 2022). We performed preliminary studies which showed that the coverages were better (especially the vertical coverage which is the most limiting) with the reference genome rather than with the MAGs. We therefore decided to focus our study only on the *B. prasinos* genome.

##
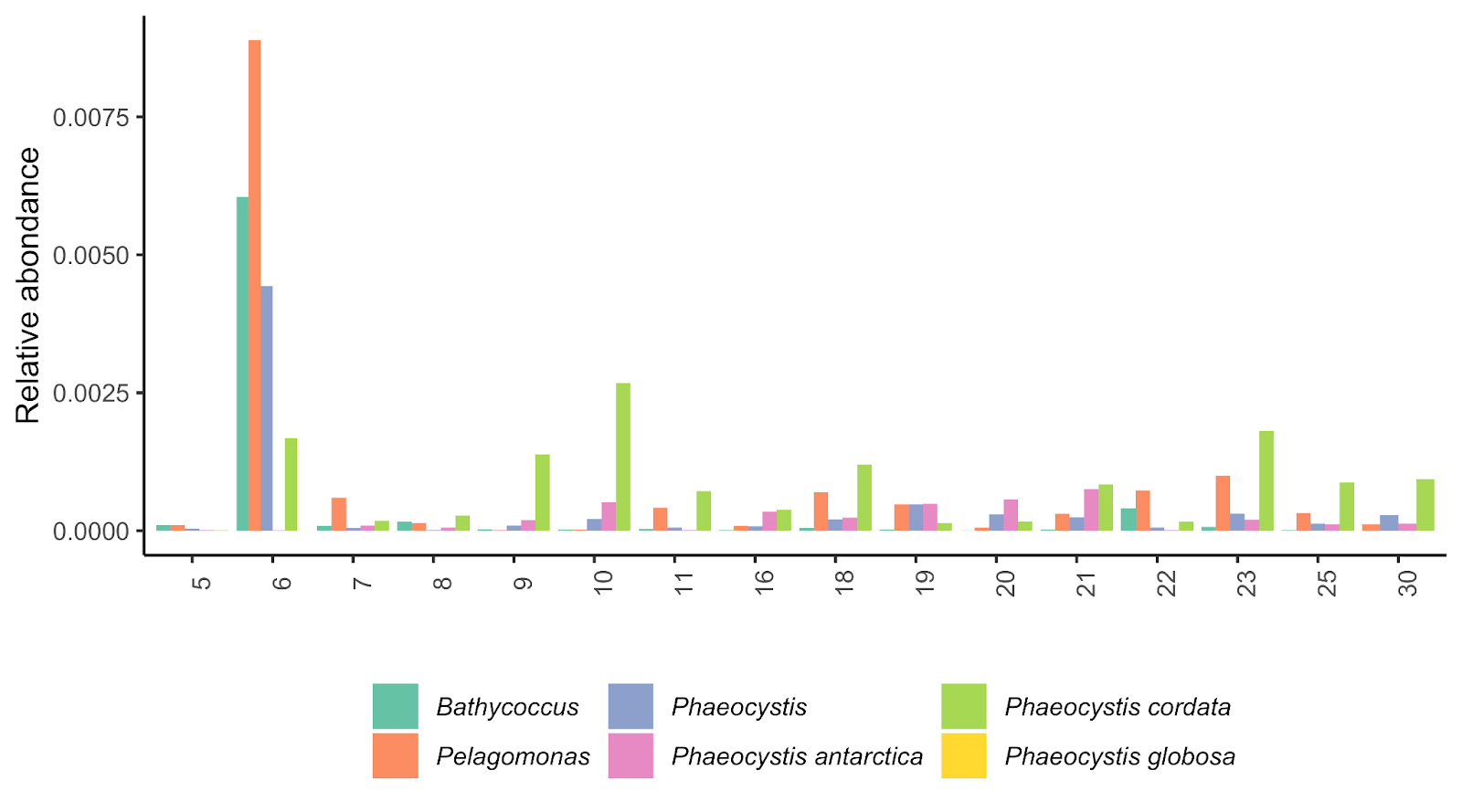


*Figure S1. Distribution of the metabarcode abundances in the Mediterranean Sea (proportion/relative abundance, Y axis) in the TO stations (X axis) (data from de Vargas et al., 2015). The selected protists belong to three distinct genera (Bathycoccus, Pelagomonas, Phaeocystis). The analysis of Bathycoccus and Pelagomonas have been done at the genus level because V9 sequences can not be distinguished for Bathycoccus species (Leconte et al., 2020), and only one species of Pelagomonas is yet described (Guiry and Guiry, 2022, Algaebase), while for Phaeocystis it has been done at the species level. Three Phaeocystis species were reported here and also the metabarcodes corresponding to P. sp. or that were only assignable to the genus rank. P. cordata shows the highest relative abundance of metabarcodes compared to the other Phaeocystis lineages in 11 of the 16 TO Mediterranean stations.*

*Table S1. Summary of the targeted species. For each species, strain used, type of reference, taxonomy and estimated cell size is provided. The table provides information for the three protists targeted by the study.*

| Species | *Bathycoccus prasinos* | *Pelagomonas calceolata* | *Phaeocystis cordata* |
| --- | --- | --- | --- |
| Strain | RCC1105 | RCC969 | RCC1383 |
| Sampling site | Mediterranean Sea (Tyrrhenian Sea) | Southern Pacific Ocean | Mediterranean Sea (Banuyls’ Bay) |
| Reference data | Genome assembly (Moreau et al., 2012) (GCA_002220235.1) 15 Mb | Transcriptome assembly (MMETSP ; Keeling et al., 2014; Johnson et al., 2019) 21 Mb | Transcriptome assembly (MMETSP ; Keeling et al., 2014; Johnson et al., 2019) 9.4 Mb |
| Taxonomy (based on Adl et al., 2019) | Chloroplastida Chlorophyta Mamiellophyceae Bathycoccus | Stramenopiles Gyrista Ochrophyta Diatomista Pelagophyceae Pelagomonadales Pelagomonas | Haptista Haptophyta Prymnesiophyceae Phaeocystales Phaeocystis |
| Estimated size from morphological studies | 1-2 µm (Eikrem and Throndsen, 1990; Moreau et al., 2012) | 1.3 x 3 µm (Andersen et al., 1993) | 3–3.5 µm long ; 3–4µm wide (Medlin and Zigone, 2007; Zingone et al., 1999) |

## References.

Adl, S.M., Bass, D., Lane, C.E., Lukes, J., Schoch, C.L., Smirnov, A., Agatha, S., *et al*. (2019). Revisions to the Classification, Nomenclature, and Diversity of Eukaryotes. J. Eukaryot. Microbiol. 66 : 4-119. https://doi.org/10.1111/jeu.12691

Andersen, R. A., Saunders, G. W., Paskind, M. P., Sexton, J. P. (1993). Ultrastructure and 18s RRNA gene sequence for pelagomonas calceolata gen. Et Sp. nov. and the description of a new algal class, the pelagophyceae classis nov. 1. J. Phycol. 29, 5. 701–715.

de Vargas, C., Audic, S., Henry, N., Decelle, J., Mahé, F., Logares, R., Lara, E., *et al*. (2015) Eukaryotic Plankton Diversity in the Sunlit Ocean. Science 348 (6237).

Delmont, T.O., Gaia, M., Hinsinger, D.D., Fremont, P., Vanni, C., Fernandez Guerra, A., et al. (2022) Functional repertoire convergence of distantly related eukaryotic plankton lineages abundant in the sunlit ocean. Cell Genomics. 2 (5), 100123.Eikrem, W., Throndsen, J. (1990). The ultrastructure of Bathycoccus gen. nov. and B. prasinos sp. nov., a non-motile picoplanktonic alga (Chlorophyta, Prasinophyceae) from the Mediterra- nean and Atlantic. Phycologia, 29, 3. 344–350.

Guiry, M.D. & Guiry, G.M. 2022. AlgaeBase. World-wide electronic publication, National University of Ireland, Galway. https://www.algaebase.org

Johnson, L. K., Alexander, H., Brown, C. T. (2019). Re-assembly, quality evaluation, and anno- tation of 678 microbial eukaryotic reference transcriptomes. GigaScience, 8, 4. giy158.

Keeling, P. J., Burki, F., Wilcox, H. M., Allam, B., Allen, E. E., Amaral-Zettler, L. A., Armbrust, E. V., *et al*. (2014). The Marine Micro- bial Eukaryote Transcriptome Sequencing Project (MMETSP) : illuminating the functional diversity of eukaryotic life in the oceans through transcriptome sequencing. PLoS biology, 12, 6. e1001889.

Leconte, J., Benites, L. F., Vannier, T., Wincker, P., Piganeau, G., & Jaillon, O. (2020). Genome resolved biogeography of mamiellales. Genes, 11(1), 66.

Medlin L., Zingone A. (2007) A taxonomic review of the genus Phaeocystis. In : van Leeuwe M.A., Stefels J., Belviso S., Lancelot C., Verity P.G., Gieskes W.W.C. (eds) Phaeocystis, major link in the biogeochemical cycling of climate-relevant elements. Springer, Dordrecht. https: //doi.org/10.1007/978-1-4020-6214-8_2 Moreau, H., Verhelst, B., Couloux, A., Derelle, E., Rombauts, S., Grimsley, N., Van Bel, M., Poulain, J., Katinka, M., Hohmann-Marriott, M. F., *et al*. (2012). Gene functionalities and ge- nome structure in Bathycoccus prasinos reflect cellular specializations at the base of the green lineage. Genome biology, 13, 8. 1–16.

Zingone, A., Chretiennot-Dinet, M-J, Lange, M., Medlin, L. (1999). Morphological and genetic characterization of Phaeocystis cordata and P. jahnii (Prymnesiophyceae), two new species from the Mediterranean Sea. J. Phycol. 35, 6. 1322–1337.

Supporting Information 2. Metagenomics analysis

Files are available on Zenodo (DOI: 10.5281/zenodo.6434681).

*Table S2.1. Metagenomic samples used for the analyses (from Carradec et al., 2018). When several samples were available, the largest one was systematically chosen. While in the exploration step, all the main eukaryotic size fractions (0.8-5 µm, 5-20 µm and 20-180 µm) were considered, the final species choice presented in the study led us to use only the metagenomic samples corresponding to the size fractions 0.8-5 µm.*

| STATION | FRACTION | SAMPLE | #READS | SIZE file.gz (Go) |
| --- | --- | --- | --- | --- |
| TARA_005 | 0.8-5 | ERR868497 | 215,288,687 | 17 |
| TARA_006 | 0.8-5 | ERR868431 | 193,698,390 | 15 |
| TARA_007 | 0.8-5 | ERR315802 | 163,300,930 | 19-20 |
| TARA_009 | 0.8-5 | ERR868407 | 185,406,539 | 16 |
| TARA_016 | 0.8-5 | ERR868412 | 163,007,547 | 13 |
| TARA_018 | 0.8-5 | ERR868393 | 168,978,860 | 14 |
| TARA_020 | 0.8-5 | ERR868504 | 167,093,109 | 13-14 |
| TARA_022 | 0.8-5 | ERR868403 | 205,419,622 | 17 |
| TARA_023 | 0.8-5 | ERR538173 | 198,522,116 | 17 |
| TARA_024 | 0.8-5 | ERR868456 | 160,573,608 | 13 |
| TARA_025 | 0.8-5 | ERR868356 | 185,404,639 | 16 |
| TARA_026 | 0.8-5 | ERR868402 | 190,669,700 | 16 |
| TARA_030 | 0.8-5 | ERR538186 | 215,014,987 | 18 |

*Table S2.2. SNPs pipeline. Steps summary and associated tools, scripts and settings.*

|  | Description | Bioinformatic tool | Settings |
| --- | --- | --- | --- |
| Step 1. Preprocessing | Remove bad quality regions | Trimmomatic (v0.33) | -threas 30; -phred33; LEADING:3; TRAILING:3; SLIDINGWINDOW:4:15 |
| Step 2. Mapping | Map metagenomic reads onto reference assembly | bwa mem (v0.7.5) | -M -t 30 |
| Step 3. Aligned reads filtering | Filter reads aligned with less than 95% identity | samtools fillmd (v1.8-3) Python (v3.7.3) | >95% identity |
|  | Remove poorly informative regions | samtools fasta PRINSEQ-lite(v0.20.4) | -lc_methoddust -lc_threshold 8 |
| Step 4. Variant calling | Generate pileup file | samtools mpileup (v1.8- 3) | -aa -uf |
|  | Compute coverages | bash |  |
|  | Call variants | bcftools call (v1.8-8) | –ploidy 1 -v -m |
| Step 5. Variants filtering | Filter variants | bcftools filter (v1.8-8) bcftools view (v1.8-8) | –exclude ’QUAL <30’ -g ^miss |
|  | Extract SNPs | bcftools view (v1.8-8) | -v snps |

*Table S2.3. Distribution of the number of SNPs in the stations (SNP) obtained after filtration steps (i.e., removing SNPs with less than 4X) and the resulting number of SNPs used to compute genetic distances (FST) between pairs of stations (pSNP).*

| Species | *B. prasinos* | *P. calceolata* | *P. cordata* |
| --- | --- | --- | --- |
| SNP_Total in all samples_ | 51,176 | 108,295 | 4,627 |
| SNP_Min - Max per sample_ | 13 - 44,467 | 195 - 79,740 | 604 - 1,867 |
| SNP_Mean per sample_ | 4,739.00 | 10,471.42 | 867.15 |
| SNP_Median per sample_ | 53 | 492.5 | 790 |
| pSNP_Min - Max per sample pair_ | 1 - 4,987 | 15 - 7,525 | 118 - 323 |
| pSNP_Mean per sample pair_ | 134.24 | 237.41 | 203.79 |
| pSNP_Median per sample pair_ | 9 | 108 | 201 |

Supporting information 3 - Geographic distances

From the geographic coordinates of the stations (PANGAEA database, Pesant et al. 2015), pairwise geographic distances were estimated. They correspond to the shortest path between each pair of stations that do not cross through the land. An example of the pairwise paths is given in the Supp. Figure 3.1. (station 12). The geographic distances are provided in the Supp. Table 3.1.

Table of geographic distances is available on [GitHub](https://github.com/opheliedasilva/popmetag).


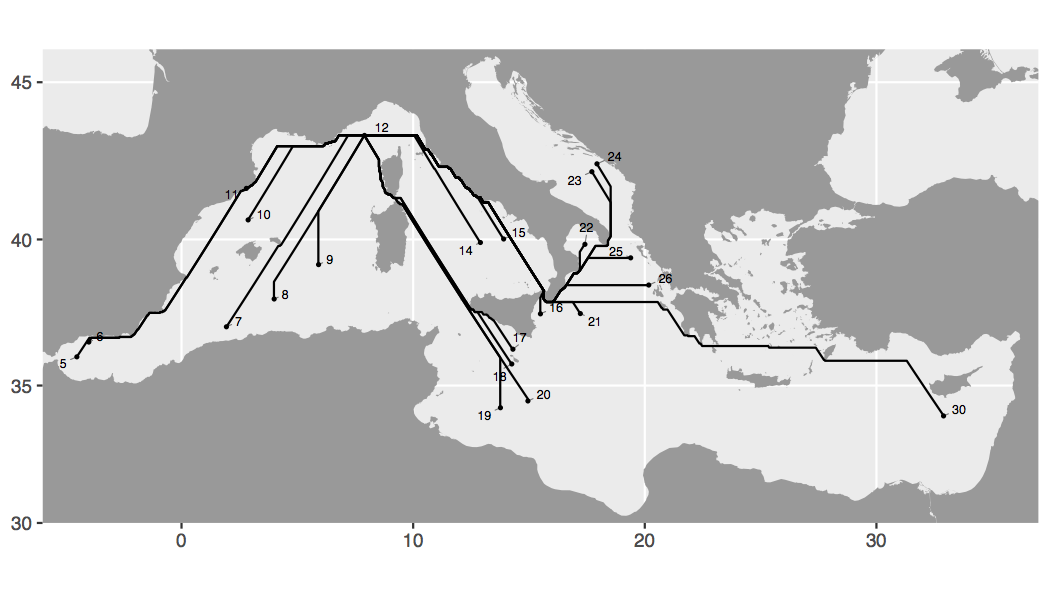


*Figure S3. Representation of the geographic distances between station 12 and all others. The calculated paths correspond to the shortest land.*

*Table S3. Geographic distances between pairwise stations*

|  | **5** | **6** | **7** | **9** | **16** | **18** | **20** | **22** | **23** | **24** | **25** | **26** | **30** |
| --- | --- | --- | --- | --- | --- | --- | --- | --- | --- | --- | --- | --- | --- |
| **5** |  |  |  |  |  |  |  |  |  |  |  |  |  |
| **6** | 77 |  |  |  |  |  |  |  |  |  |  |  |  |
| **7** | 636 | 560 |  |  |  |  |  |  |  |  |  |  |  |
| **9** | 1102 | 1025 | 473 |  |  |  |  |  |  |  |  |  |  |
| **16** | 1969 | 1892 | 1334 | 989 |  |  |  |  |  |  |  |  |  |
| **18** | 1842 | 1765 | 1207 | 933 | 241 |  |  |  |  |  |  |  |  |
| **20** | 1984 | 1908 | 1349 | 1076 | 354 | 165 |  |  |  |  |  |  |  |
| **22** | 2253 | 2176 | 1622 | 1240 | 341 | 557 | 663 |  |  |  |  |  |  |
| **23** | 2595 | 2518 | 1964 | 1581 | 683 | 899 | 975 | 380 |  |  |  |  |  |
| **24** | 2615 | 2538 | 1983 | 1601 | 703 | 919 | 995 | 400 | 34 |  |  |  |  |
| **25** | 2365 | 2288 | 1734 | 1351 | 453 | 669 | 690 | 198 | 354 | 374 |  |  |  |
| **26** | 2379 | 2302 | 1748 | 1366 | 467 | 683 | 703 | 321 | 475 | 494 | 122 |  |  |
| **30** | 3624 | 3548 | 2989 | 2715 | 1771 | 1798 | 1680 | 1737 | 1882 | 1901 | 1541 | 1420 |  |

Supporting information 4 - Environmental distances

Table of environmental distances is available on [GitHub](https://github.com/opheliedasilva/popmetag).

*Table S4. Environmental distances computed as euclidean distances between pairwise stations in the PCA space.*

|  | **5** | **6** | **7** | **9** | **16** | **18** | **20** | **22** | **23** | **24** | **25** | **26** | **30** |
| --- | --- | --- | --- | --- | --- | --- | --- | --- | --- | --- | --- | --- | --- |
| **5** |  |  |  |  |  |  |  |  |  |  |  |  |  |
| **6** | 0.72 |  |  |  |  |  |  |  |  |  |  |  |  |
| **7** | 5.36 | 5.74 |  |  |  |  |  |  |  |  |  |  |  |
| **9** | 3.98 | 4.19 | 2.98 |  |  |  |  |  |  |  |  |  |  |
| **16** | 6.61 | 6.81 | 2.82 | 2.8 |  |  |  |  |  |  |  |  |  |
| **18** | 5.94 | 6.21 | 2.27 | 2.17 | 0.93 |  |  |  |  |  |  |  |  |
| **20** | 6.68 | 6.92 | 2.87 | 2.8 | 0.58 | 0.78 |  |  |  |  |  |  |  |
| **22** | 4.81 | 4.88 | 4.82 | 1.95 | 3.69 | 3.32 | 3.62 |  |  |  |  |  |  |
| **23** | 4.95 | 4.93 | 6.21 | 3.27 | 5.22 | 4.84 | 5.13 | 1.54 |  |  |  |  |  |
| **24** | 4.73 | 4.74 | 5.98 | 3.05 | 5.1 | 4.67 | 4.99 | 1.41 | 0.31 |  |  |  |  |
| **25** | 5.34 | 5.46 | 3.69 | 1.49 | 2.12 | 1.88 | 2.12 | 1.59 | 3.11 | 2.99 |  |  |  |
| **26** | 6.17 | 6.28 | 3.4 | 2.33 | 1.1 | 1.46 | 1.4 | 2.87 | 4.37 | 4.28 | 1.32 |  |  |
| **30** | 7.31 | 7.24 | 4.63 | 4.21 | 2.83 | 3.53 | 3.39 | 4.78 | 6.07 | 6.05 | 3.51 | 2.45 |  |

Supporting information 5 - Oceanographic distances

The Lagrangian data used are from two biophysical models. The first model is the one established by Berline et al. (2014). The Mediterranean Sea was cut into a 50km cell grid and the velocity fields from satellite data (2007-2011) were used to derive basin-wide trajectories. Here, the trajectories correspond to the 0 and 50m depths. When station pairs are connected in a time > 365 days (i.e., Lagrangian particle advection time), the MCT is estimated using the MCT between intermediate points. This indirect estimate leads to less accurate values.

The second model, from Ser-Giacomi et al. (2015a), adopts the Lagrangian flow network approach in which networks describing water transport across the Mediterranean Sea are built. Each network is composed of a set of oceanic nodes representing small, equal-sized subregions of the Mediterranean Sea surface (Rossi et al., 2015). Links and weights between such sets of nodes quantify water parcels exchanges driven by ocean currents over a time-interval. By normalizing the network adjacency matrices, probabilities of connection between each pair of nodes are derived (Ser-Giacomi et al., 2015b, Ser-Giacomi et al., 2017).

To construct the network, we use a reference horizontal flow field produced by an operational data-assimilating ocean model (Simoncelli et al., 2019). Each network node is filled with 625 Lagrangian particles and its horizontal size is 771.9 km². Lagrangian trajectories are then reconstructed using a Runge-Kutta 4 integration scheme with a time-step of 6 hours. In such a way we build networks representing the average subsurface physical transport across 2005-2009 for a duration of 90, 180 and 360 days. Finally, connection probabilities between pairs of Tara sampling stations are calculated as the normalized weights of the associated pairs of network nodes.

**References.**

Berline, L., Rammou, A.M., Doglioli, A., Molcard, A., and Petrenko., A. (2014) A Connectivity-Based Eco-Regionalization Method of the Mediterranean Sea. PloS One 9 (11): e111978.

Rossi, V., Ser‐Giacomi, E., López, C., and Hernández‐García, E. (2014). Hydrodynamic provinces and oceanic connectivity from a transport network help designing marine reserves. Geophysical Research Letters, 41(8), 2883-2891.

Ser-Giacomi, E., Rossi, V., López, C., and Hernández-Garcı́a, E. (2015a) Flow Networks: A Characterization of Geophysical Fluid Transport. Chaos 25 (3): 036404.

Ser-Giacomi, E., Vasile, R., Hernandez-Garcia, E., and López, C. (2015b). Most probable paths in temporal weighted networks: An application to ocean transport. Physical review E, 92(1), 012818.

Ser-Giacomi, E., Rodríguez-Méndez, V., López, C., and Hernández-García, E. (2017). Lagrangian Flow Network approach to an open flow model. The European Physical Journal Special Topics, 226(9), 2057-2068.

Simoncelli, S., Fratianni, C., Pinardi, N., Grandi, A., Drudi, M., Oddo, P., and Dobricic, S. (2019). Mediterranean sea physical reanalysis (CMEMS MED-physics)[data set]. Copernicus Monitoring Environment Marine Service (CMEMS).

Tables of oceanographic distances are available on [GitHub](https://github.com/opheliedasilva/popmetag).

*Table S5.1. Oceanographic distances computed as mean connection time. The rows correspond to the departure stations and the columns to the arrival stations.*

|  | **5** | **6** | **7** | **9** | **16** | **18** | **20** | **22** | **23** | **24** | **25** | **26** | **30** |
| --- | --- | --- | --- | --- | --- | --- | --- | --- | --- | --- | --- | --- | --- |
| **5** |  |  |  |  |  |  |  |  |  |  |  |  |  |
| **6** | 46 |  |  |  |  |  |  |  |  |  |  |  |  |
| **7** | 105 | 100 |  |  |  |  |  |  |  |  |  |  |  |
| **9** | 193 | 192 | 132 |  |  |  |  |  |  |  |  |  |  |
| **16** | 247 | 244 | 202 | 191 |  |  |  |  |  |  |  |  |  |
| **18** | 240 | 232 | 200 | 199 | 115 |  |  |  |  |  |  |  |  |
| **20** | 285 | 283 | 256 | 226 | 179 | 120 |  |  |  |  |  |  |  |
| **22** | 276 | 264 | 250 | 229 | 138 | 208 | 212 |  |  |  |  |  |  |
| **23** | 311 | 316 | 295 | 270 | 160 | 262 | 284 | 152 |  |  |  |  |  |
| **24** | 312 | 316 | 296 | 269 | 158 | 263 | 278 | 149 | 50 |  |  |  |  |
| **25** | 301 | 299 | 273 | 254 | 148 | 193 | 240 | 115 | 85 | 81 |  |  |  |
| **26** | 296 | 288 | 256 | 245 | 126 | 226 | 240 | 126 | 98 | 92 | 56 |  |  |
| **30** | 544 | 546 | 412 | 304 | 264 | 281 | 265 | 291 | 290 | 292 | 273 | 259 |  |

*Table S5.2. Oceanographic distances computed as probabilities of connection. The rows correspond to the departure stations and the columns to the arrival stations.*

|  | **5** | **6** | **7** | **9** | **16** | **18** | **20** | **22** | **23** | **24** | **25** | **26** | **30** |
| --- | --- | --- | --- | --- | --- | --- | --- | --- | --- | --- | --- | --- | --- |
| **5** |  |  |  |  |  |  |  |  |  |  |  |  |  |
| **6** | 6.76x10^-4^ |  |  |  |  |  |  |  |  |  |  |  |  |
| **7** | 1.16x10^-3^ | 1.48x10^-3^ |  |  |  |  |  |  |  |  |  |  |  |
| **9** | 1.00x10^-3^ | 1.05x10^-3^ | 1.55x10^-3^ |  |  |  |  |  |  |  |  |  |  |
| **16** | 5.55x10^-5^ | 4.92x10^-5^ | 1.07x10^-4^ | 2.04x10^-4^ |  |  |  |  |  |  |  |  |  |
| **18** | 3.19x10^-4^ | 2.83x10^-4^ | 5.86x10^-4^ | 8.95x10^-4^ | 2.89x10^-4^ |  |  |  |  |  |  |  |  |
| **20** | 1.48x10^-4^ | 1.28x10^-4^ | 3.09x10^-4^ | 5.47x10^-4^ | 2.85x10^-4^ | 2.33x10^-3^ |  |  |  |  |  |  |  |
| **22** | 8.79x10^-6^ | 7.41x10^-6^ | 2.28x10^-5^ | 5.39x10^-5^ | 1.70x10^-4^ | 1.61x10^-4^ | 8.32x10^-5^ |  |  |  |  |  |  |
| **23** | 4.06x10^-7^ | 3.3x10^-7^ | 1.32x10^-6^ | 3.98x10^-6^ | 1.02x10^-4^ | 1.38x10^-5^ | 1.04x10^-5^ | 5.38x10^-4^ |  |  |  |  |  |
| **24** | 6.48x10^-7^ | 5.37x10^-7^ | 2.16x10^-6^ | 6.44x10^-6^ | 1.62x10^-4^ | 2.20x10^-5^ | 1.21x10^-5^ | 5.60x10^-4^ | 9.07x10^-3^ |  |  |  |  |
| **25** | 1.33x10^-5^ | 1.143x10^-5^ | 3.30x10^-5^ | 7.64x10^-5^ | 1.32x10^-3^ | 2.27x10^-4^ | 1.27x10^-4^ | 2.38x10^-3^ | 1.14x10^-3^ | 1.58x10^-3^ |  |  |  |
| **26** | 1.62x10^-5^ | 1.43x10^-3^ | 3.43x10^-5^ | 7.11x10^-5^ | 1.42x10^-3^ | 2.26x10^-4^ | 1.20x10^-4^ | 1.20x10^-3^ | 3.83x10^-4^ | 1.02x10^-3^ | 5.93x10^-3^ |  |  |
| **30** | 2.05x10^-7^ | 1.72x10^-7^ | 9.48x10^-7^ | 4.42x10^-6^ | 4.24x10^-5^ | 5.65x10^-5^ | 4.66x10^-5^ | 8.73x10^-6^ | 2.76x10^-6^ | 3.19x10^-6^ | 3.27x10^-5^ | 6.76x10^-5^ |  |

Supporting information 6 – Contamination by closely related species : *B. prasinos* (clade BI) and *B. calidus* (clade BII)

Two *Bathycoccus* clades/ecotypes are currently recognised. *Bathycoccus* Clade I (BI) is named *B. prasinos* and was isolated in the Mediterranean Sea. It corresponds to RCC1105, the dataset we used. *Bathycoccus* Clade II (BII), named *B. calidus*, is represented by the Indian Ocean isolates RCC715 and RCC716, described in Bachy et al. (2021) The BII corresponds also to the SAG assembly named TOSAG39-1 (Vannier et al., 2016).

The BI (RCC1105) and BII (TOSAG39-1) are two different ecotypes as demonstrated in the global metagenomic survey performed by Vannier et al. (2016). Even if their distribution may overlap, these two clades rarely co-occur as they occupy distinct ecological niches. Especially in the Mediterranean Sea, BII (*B. calidus*, TOSAG39-1) seems less abundant than BI (*B. prasinos*, RCC1105) in the Tara Oceans surface waters (Vannier et al., 2016). Indeed, BII is associated with warmer and saltier waters, as well as deeper environments in comparison to BI (Monier et al., 2013, Vannier et al., 2016, Limardo et al., 2017).

Vannier et al. (2016) also identified large genomic differences between the two clades (*i.e.*, only 26 genes are highly conserved, with >99% identity) and claimed that they can be distinguished during the read recruitment step with the 95% identity threshold.

The identity threshold cannot guarantee that all recruited reads belong to the target species, that may artificially increase the SNPs number. Because the conserved genomic regions are likely to recruit more reads (those corresponding to BI and BII), all SNPs with vertical coverage higher than µ+2σ (µ and σ being respectively the mean and standard deviation of the vertical coverage for each metagenomic sample) have been filtered. This step aims to remove noise due to the read recruitment of close species (here BII) (Madoui et al., 2017; Arif et al., 2019; Laso-Jadart et al., 2020a,b, 2021, Leconte et al., 2021)

To estimate whether the presence of the BII clade could impact our results, we mapped the same TO metagenomic samples on TOSAG39-1 genome using the same parameters (95% sequence identity).

The reads aligned on TOSAG39-1 were then mapped on RCC1105, and generated 352 SNPs. In comparison with the 51176 SNPs used for the RCC1105 analysis, only 170 SNPs remained and 182 SNPs were removed by the µ+2σ vertical filter (Figure S6).

These 170 SNPs for our study focused on RCC1105 can be considered as false positives (i.e. due to reads that really belong to TOSAG39-1/BII) or as true positives (*i.e.*, reads from RCC1105/BI that were recruited as belonging to TOSAG39-1/BII, for instance in a highly conserved region).

In conclusion, recruiting reads from BII could lead to detecting false positive SNPs (i.e., interspecies variability) when analysing BI. These false positives could influence the genomic distance between stations. Pair of samples with BII would tend to get lower genomic differentiation than with other samples due to these false positive SNPs. On the contrary, if BII is present in only one of the two samples, the pairwise genomic differentiation would be overestimated.

However, as these potential false positives only correspond to 0.3% of the SNPs we are rather confident that the genomic distances are not strongly impacted by them.

To summarize, based on the four points mentioned above (*i.e.*, distinct niches, identity threshold, vertical filtering and low SNP overlap), we assumed that the contamination by BII and its impact on our results are limited.


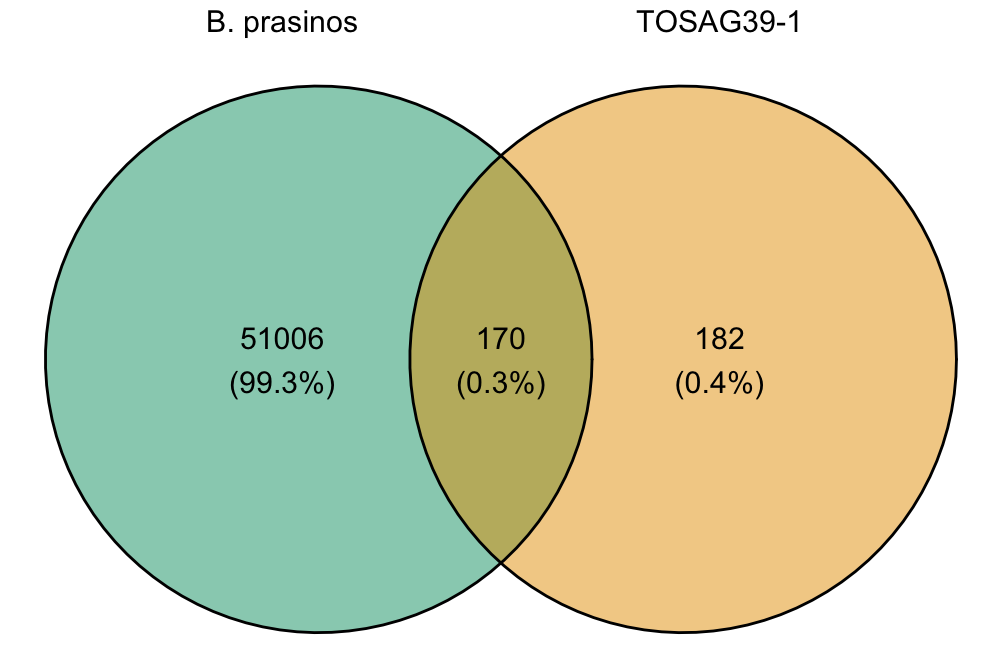


Figure S6. Number of SNPs detected along the RCC1105 genome (BI, *B. prasinos*) and the TOSAG39-1 genome (BII, *B. calidus*).

**References.**

Arif, M., Gauthier, J., Sugier, K., Iudicone, D., Jaillon, O., Wincker, P., Peterlongo, P., & Madoui, M. A. (2019). Discovering millions of plankton genomic markers from the Atlantic Ocean and the Mediterranean Sea. Molecular ecology resources, 19(2), 526–535. <https://doi.org/10.1111/1755-0998.12985>

Bachy, C., Yung, C.C.M., Needham, D.M., Gazitúa, M.C., Roux, S., Limardo, A.J., Choi, C.J., Jorgens, D.M., Sullivan, M.B., Worden, A.Z., 2021. Viruses infecting a warm water picoeukaryote shed light on spatial co-occurrence dynamics of marine viruses and their hosts. The ISME Journal 1–19. <https://doi.org/10.1038/s41396-021-00989-9>

Laso-Jadart, R., Ambroise, C., Peterlongo, P., & Madoui, M. A. (2020). metaVaR: Introducing metavariant species models for reference-free metagenomic-based population genomics. PloS one, 15(12), e0244637. <https://doi.org/10.1371/journal.pone.0244637>

Laso-Jadart, R., Sugier, K., Petit, E., Labadie, K., Peterlongo, P., Ambroise, C., Wincker, P., Jamet, J. L., & Madoui, M. A. (2020). Investigating population-scale allelic differential expression in wild populations of Oithona similis (Cyclopoida, Claus, 1866). Ecology and evolution, 10(16), 8894–8905. <https://doi.org/10.1002/ece3.6588>

Laso-Jadart, R., O’Malley, M., Sykulski, A. M., Ambroise, C., & Madoui, M. A. (2021). How marine currents and environment shape plankton genomic differentiation: a mosaic view from Tara Oceans metagenomic data. *Biorxiv*. <https://doi.org/10.1101/2021.04.29.441957>

Leconte, J., Timsit, Y., Delmont, T. O., Lescot, M., Piganeau, G., Wincker, P., & Jaillon, O. (2021). Equatorial to Polar genomic variability of the microalgae Bathycoccus prasinos. *bioRxiv*. <https://doi.org/10.1101/2021.07.13.452163>

Madoui, M. A., Poulain, J., Sugier, K., Wessner, M., Noel, B., Berline, L., Labadie, K., Cornils, A., Blanco-Bercial, L., Stemmann, L., Jamet, J. L., & Wincker, P. (2017). New insights into global biogeography, population structure and natural selection from the genome of the epipelagic copepod Oithona. Molecular ecology, 26(17), 4467–4482. <https://doi.org/10.1111/mec.14214>

Vannier, T., Leconte, J., Seeleuthner, Y., Mondy, S., Pelletier, E., Aury, J. M., *et al.* (2016). Survey of the green picoalga Bathycoccus genomes in the global ocean. Scientific reports, 6(1), 1-11.

**Supporting information 7 - The use of genomic reference *versus* transcriptomic reference in the case of *B. prasinos***

SNPs, allelic frequencies and so, *F_ST_* are not uniformly distributed along genomes, due to lower constraints on non-coding regions (Piganeau et al., 2009), that may indicate genes under selection (Narum and Hess, 2011). Using transcriptomics (coding sequences) rather than genomics (coding and non-coding sequences) could lead to detecting less SNPs and to increase the proportion of neutral SNPs (*i.e.*, leading to an amino acid change) versus non-neutral SNPs. To accurately quantify genetic differentiation, genome-wide approaches should ideally be conducted. Therefore, we have favored genomes as reference when available, even if we decided to include transcriptomes in our analysis to overcome the lack of references.

In our case, as other Mamiellales, *Bathycoccus prasinos* is characterized by a high gene density (Moreau et al., 2012). Due to this compact genome, we hypothesized a good correlation between genome and transcriptome. So, although in the general case discrepancies between genome-wide and transcriptome-wide approaches can be highlighted, we can assume they are here limited.

A *B. prasinos* transcriptome is currently available (MMETSP1399 corresponding to the strain CCMP1898, Vannier et al., 2016; reassembly is available in the METDB database <http://metdb.sb-roscoff.fr/metdb/assembly/36/>). We performed a BUSCO analysis for both and, even if MMETSP1399 showed relatively good metrics, it is outperformed by RCC1105 (Table S7), which argues in favor of using the genome rather than the transcriptome as reference for *B. prasinos*.

In order to better measure the potential impact of using the transcriptome of *B. prasinos* rather than the genome, we performed analysis with the decontaminated MMETSP reference (available at <https://metdb.sb-roscoff.fr/metdb/assembly/36/>). Vertical and horizontal coverages are very close for the two reference types but, even if comparable, the number of SNPs is higher with the genome (total of 51,176 and 43,073 SNPs for the genome and the transcriptome, respectively). Moreover, the best statistical model still is the one with the environment as a predictive variable, but the relationship is not significant anymore (p-value = 0.12). Given the low predictive power of our models highlighted in our manuscript, this is not surprising. We can hypothesize that the genome-wide approach by allowing a clearer discrimination of genomic differentiation, leads to the identification of the possible process driving it.

Table S7. Summary of the BUSCO analysis performed on the genome (RCC1105, GenBank assembly accession: GCA_002220235.1) and the transcriptome (MMETSP1399, Meddbiid: METDB_00036) of *B. prasinos*, using the dataset chlorophyta_odb10 (number of genomes: 16, number of BUSCOs: 1519). All metrics are better for RCC1105 rather than for MMETSP1399.

|  | **RCC1105**  **(genome)** | **MMETSP1399 (transcriptome)** |
| --- | --- | --- |
| **Complete BUSCOs** | 97.1% | 93.2% |
| **Complete and single-copy BUSCOs** | 96.7% | 83% |
| **Complete and duplicated BUSCOs** | 0.4% | 10.2% |
| **Fragmented BUSCOs** | 0.7% | 2% |
| **Missing BUSCOs** | 2.2% | 4.8% |
| **Total BUSCO groups searched** | 1519 | 1519 |

**References**

Moreau, H., Verhelst, B., Couloux, A., Derelle, E., Rombauts, S., Grimsley, N., *et al*. (2012). Gene functionalities and genome structure in Bathycoccus prasinos reflect cellular specializations at the base of the green lineage. *Genome biology*, *13*(8), 1-16. <https://doi.org/10.1186/gb-2012-13-8-r74>

Narum, S. R., & Hess, J. E. (2011). Comparison of FST outlier tests for SNP loci under selection. *Molecular ecology resources*, *11*, 184-194. <https://doi.org/10.1111/j.1755-0998.2011.02987.x>

Piganeau, G., Vandepoele, K., Gourbiere, S., Van de Peer, Y., & Moreau, H. (2009). Unravelling cis-regulatory elements in the genome of the smallest photosynthetic eukaryote: phylogenetic footprinting in Ostreococcus. *Journal of Molecular Evolution*, *69*(3), 249-259. <https://doi.org/10.1007/s00239-009-9271-0>),

Vannier, T., Leconte, J., Seeleuthner, Y., Mondy, S., Pelletier, E., Aury, J. M., *et al.* (2016). Survey of the green picoalga Bathycoccus genomes in the global ocean. Scientific reports, 6(1), 1-11. <https://doi.org/10.1038/srep37900>

Supporting information 8 - Overview of the study


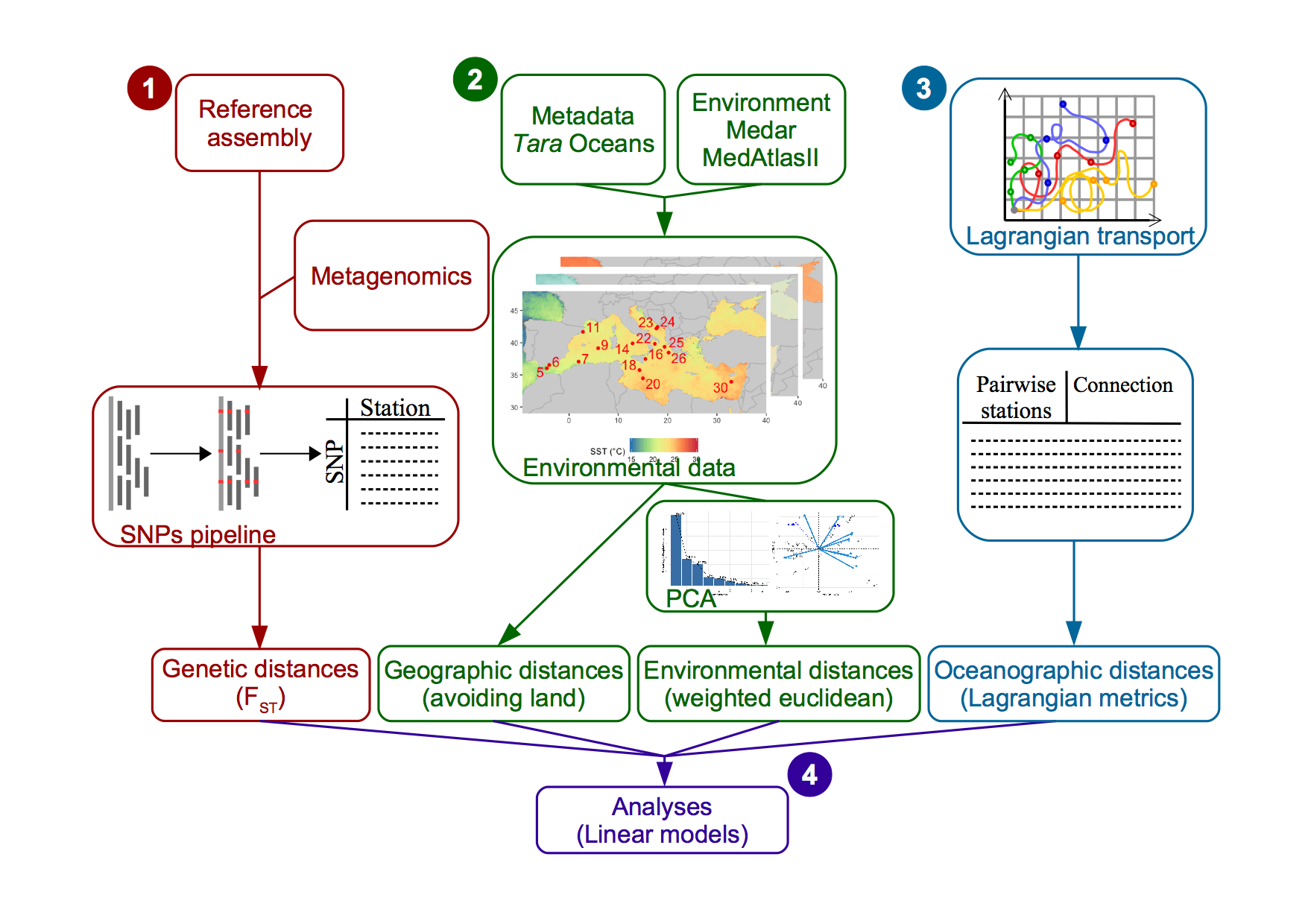


*Figure S8. Analysis strategy for population metagenomics of marine protists detailed in 4 parts. The colours of the boxes indicate the genomic (red), geographic and environmental (green) or oceanographic (blue) parts of the study. PCA: Principal Component Analysis. SNP: Single Nucleotide Polymorphisms.*

Codes for each analyse is provided on [GitHub](https://github.com/opheliedasilva/popmetag) :

- main pipeline for SNPs analysis (red, top) in **pipeline** repository
- Rcode for the genomic analysis (red, bottom): 0_preprocessing_fst.R
- Rcode for geographical analysis (green, left ): 0_preprocessing_geo.R
- Rcode for the environmental analysis (green, right): 0_preprocessing_env.R and 1_analysis_environment.R
- Rcode for oceanographic analysis (blue): 0_preprocessing_circu_mct.R and 0_preprocessing_circu_proba_cnx.R
- Rcode for statistical analysis (purple): 1_analysis_linear_models.R
